# IFNγ-induced antigen loss in chimeric antigen receptor-T cell therapy

**DOI:** 10.3389/fimmu.2026.1772472

**Published:** 2026-03-17

**Authors:** Miao Cao, Jasmine Alvarez, Ramkrishna Mitra, Michael Xu, Trevor R. Baybutt, Zhengyang Sun, Oluwatobiloba Taylor, Allison S. Doermann, Ross E. Staudt, Scott A. Waldman, Adam E. Snook

**Affiliations:** 1 Department of Pharmacology, Physiology, and Cancer Biology, Thomas Jefferson University, Philadelphia, PA, United States; 2 Department of Surgery, Thomas Jefferson University, Philadelphia, PA, United States

**Keywords:** antigen escape, CAR-T cell, colorectal cancer, GUCY2C, IFNγ, tumor microenvironment

## Abstract

**Introduction:**

FDA-approved chimeric antigen receptor (CAR)-expressing T cell therapies (CARTs) have revolutionized the treatment of blood cancers. Yet none have been successful for "solid" tumors, such as colorectal cancer (CRC), the 2nd leading cause of cancer deaths. Guanylyl cyclase C (GUCY2C) has emerged as a clinical-stage target for CART and bispecific T-cell engager (BiTE) therapies in CRC. IFNγ has been canonically recognized as beneficial for the effector functions of T cells by enhancing antigen processing and HLA presentation and is essential for CART targeting of solid malignancies by inducing adhesion molecule expression for synapse stabilization.

**Methods:**

Using *in vitro* co-culture systems, conditioned media experiments, cytokine screening, pharmacologic inhibition, CRISPR-Cas9 knockout, and transcriptomic analyses, we investigated mechanisms of GUCY2C antigen loss in CRC cells exposed to activated CART cells.

**Results:**

We identified a novel antigen loss mechanism that limits the efficacy of CART in CRC, in which IFNγ secreted by activated CART cells causes bystander cancer cells to lose GUCY2C. This previously unexplored antigen loss mechanism is mediated through IFNγ receptor, JAK, and cellular stress signaling pathways. This mechanism of antigen loss can be rescued with anti-IFNγ neutralizing antibody, the JAK inhibitor ruxolitinib, or 4-phenylbutyrate (an ER stress reliever).

**Discussion:**

We revealed a negative effect of IFNγ that uniquely interferes with immunotherapies targeting native surface antigens, such as CART and BiTE therapies, which may be reversed by disrupting stress signaling pathways to enhance solid tumor CART and BiTE immunotherapies.

## 1 Introduction

Immunotherapy has been established as the 4^th^ pillar of cancer care, in addition to surgery, chemotherapy, and radiotherapy (1, 2). Immune checkpoint blockade (ICB) has significantly impacted outcomes in lung cancer and melanoma, but its efficacy in colorectal cancer (CRC) is limited to only a small percentage of patients with mismatch repair-deficient (dMMR) or microsatellite instable (MSI) CRC (3–5). Several bispecific T-cell engagers (BiTEs), which possess arms that bind to surface antigens on cancer cells and CD3 on T cells, have been approved to treat hematological cancers (6–8). Moreover, FDA-approved chimeric antigen receptor (CAR)-expressing T cells (CARTs) targeting CD19 and B-cell maturation antigen (BCMA) have remarkably improved outcomes for patients with relapsed/refractory hematologic malignancies (9–11). However, no CARTs for solid malignancies have achieved efficacy and safety profiles in clinical trials to obtain FDA approval.

Several antigens have been explored as targets for CART and BiTE immunotherapies in CRC (12). Human epidermal growth factor-2 (HER2), a receptor tyrosine kinase upstream of many tumorigenic signaling pathways, was among the first (13). The HER2-directed therapeutic monoclonal antibody (mAb), trastuzumab, has a well-established survival benefit and safety profile in patients with breast and gastric cancers (12, 14–16). In 2024, the antibody-drug conjugate (ADC) trastuzumab-deruxtican was approved for HER2-amplified solid tumors, including CRC (17, 18). While Her2CART has produced severe toxicity in patients (19), it remains a very active focus of research for CRC CART with efforts to affinity-tune the CAR to target HER2^high^ tumor cells and spare HER2^low^ normal tissues (20–22) or employ hypoxia-inducible CARs, a clinically-validated strategy (23) to spare HER2-expressing normal tissues (24). Another promising target under clinical investigation is cadherin-17 (CDH17). This newly discovered cell-junction antigen is differentially organized in normal and tumor tissues, allowing CDH17-directed CART (CDH17CART) to be safe and efficacious in preclinical models (25). CDH17CART is now being evaluated in a phase I/II clinical trial (NCT06055439) in CRC patients. Further, guanylate cyclase C (GUCY2C) is a well-established antigen for CRC immunotherapies. Indeed, a phase I clinical trial established the safety and immunogenicity of adenovirus-based GUCY2C vaccination in early-stage CRC patients (26). A GUCY2C-specific BiTE was developed by Pfizer and advanced to clinical testing (27). In addition, we have designed GUCY2C-directed CART (GucyCART), which has promising safety and efficacy profiles in preclinical models with high antigen expression (28–30). GUCY2C-directed CART therapies have been advanced into patients in China and the U.S. with mixed safety and efficacy results (31–34).

CART failure in solid tumors may reflect multiple factors, including poor CART expansion, persistence, and tumor homing, as well as antigen heterogeneity and escape. Indeed, antigen escape, referring to the partial or complete loss of antigen after treatment, is a known limitation of CART therapy in patients. Approximately 30-70% of patients have presented with antigen escape across multiple studies after CD19- or BCMA-CART therapy (35–40). Similarly, antigen escape has been observed in multiple CART clinical trials for solid malignancies, including those targeting EGFRvIII (41), IL13Rα2 (42), mesothelin (43), and HER2 (20). Importantly, all known antigen loss mechanisms, including genetic mutation, allelic loss, and alternative mRNA splicing, are dependent on the direct selection and/or survival pressure exerted by CARTs (35, 36, 38, 40). In contrast, here we identified an unexpected induction of GUCY2C, CDH17, and HER2 antigen loss in bystander CRC cells, mediated by IFNγ and canonical IFNγR/JAK signaling, but not by other CART-derived cytokines. Antigen loss reflected the induction of stress signals by IFNγ, which could be reversed by pharmacologically targeting stress. Together, these studies identify a potential mechanism underlying CART failure in solid tumors, which, in contrast to genetic antigen loss, might be reversed by targeted combination therapy.

## 2 Results

### 2.1 GucyCART induces GUCY2C loss in multiple CRC cell lines

**Figure 1:**
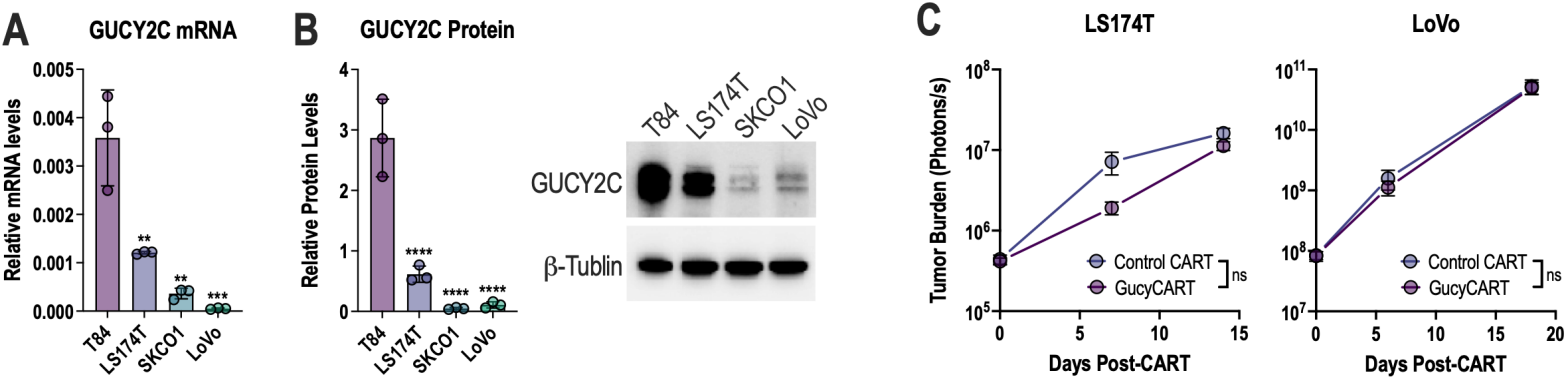
GucyCART has limited efficacy in low antigen models. **(A, B)** GUCY2C RNA **(A)** and protein **(B)** levels in various CRC models relative to the T84 cell line. **p < 0.01, ***p < 0.001, ****p < 0.0001, One-way ANOVA comparing each cell line to T84 cells. Each data point in **(A, B)** represents the average from a biological replicate. **(C)** Tumor burden of LS174T (N = 7) or LoVo (N = 5) cells injected i.p. in NSG mice before i.v. treatment with 5x10^6^ control or GucyCART cells. Areas under the curve (AUCs) were quantified, and an unpaired T-test was used for comparisons (ns = p > 0.05). Figure schematics were generated using BioRender.com.

**Figure 2:**
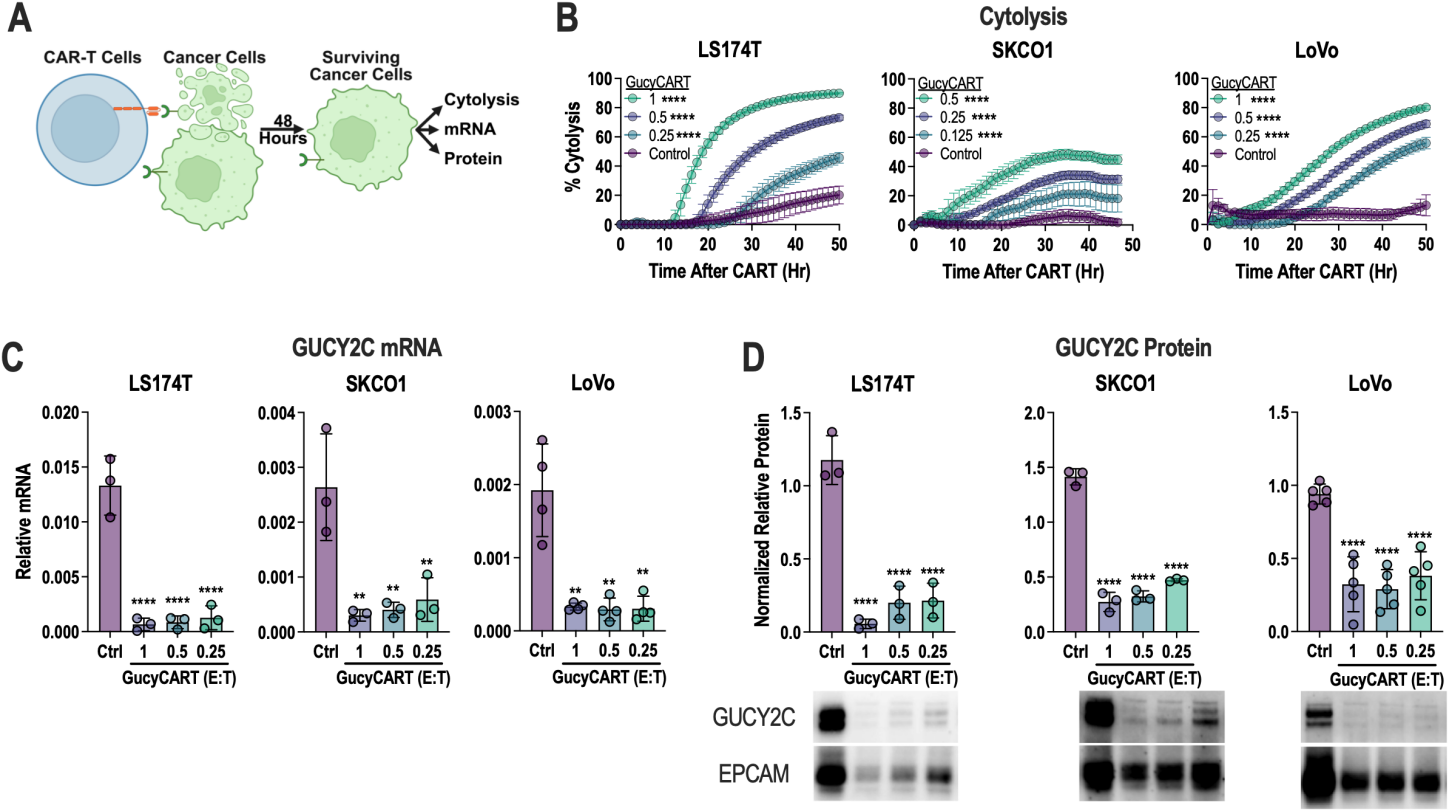
GucyCART induces GUCY2C loss in multiple low antigen models. **(A)** LS174T, SKCO1, and LoVo CRC cells were exposed to GucyCART for 48 hours. **(B)** Cytolysis kinetics were quantified over the 48 hour co-culture. AUCs were calculated for each condition, and one-way ANOVA was used to compare GucyCART and control CART at each E:T ratio. Each data point in **(B)** represents the mean ± SD from n ≥ 3 technical replicates in a single experiment that is representative of 3–5 experiments; ****p < 0.0001. **(C, D)** Following 48 hours of cytolysis, remaining cells were collected, and GUCY2C mRNA **(C)** and protein **(D)** were quantified relative to the epithelia-specific housekeeping control EPCAM. Each data point represents the average of biological replicates in separate experiments (N = 3–5 experiments). One-way ANOVA was used to compare each E:T of GucyCART to control CART; **p < 0.01, ***p < 0.0001. Figure schematics were generated using BioRender.com.

Previous studies demonstrated that GucyCART effectively treats intraperitoneal CRC tumors established in mice with T84 cells that express abundant GUCY2C (28–30). However, in intraperitoneal models of LS174T and LoVo tumors, which express lower levels of GUCY2C mRNA (**Figure 1A**) and protein (**Figure 1B**), GucyCART has limited efficacy (**Figure 1C**). *In vitro*, GucyCART lyse all cell lines with moderate and low antigen expression (**Figures 2A, B**). However, consistent with *in vivo* failure in LS174T and LoVo models (**Figure 1C**), we observed slow and incomplete cytolysis at low effector-to-target (E:T) ratios (**Figure 2B**). Therefore, we hypothesized that *in vitro* and *in vivo* failure may reflect selection of GUCY2C-loss variants. Indeed, CRC cells that persisted following cytolysis had substantially lower GUCY2C mRNA and protein levels (**Figures 2C, D**).

### 2.2 GUCY2C loss occurs in bystander CRC cells

**Figure 3:**
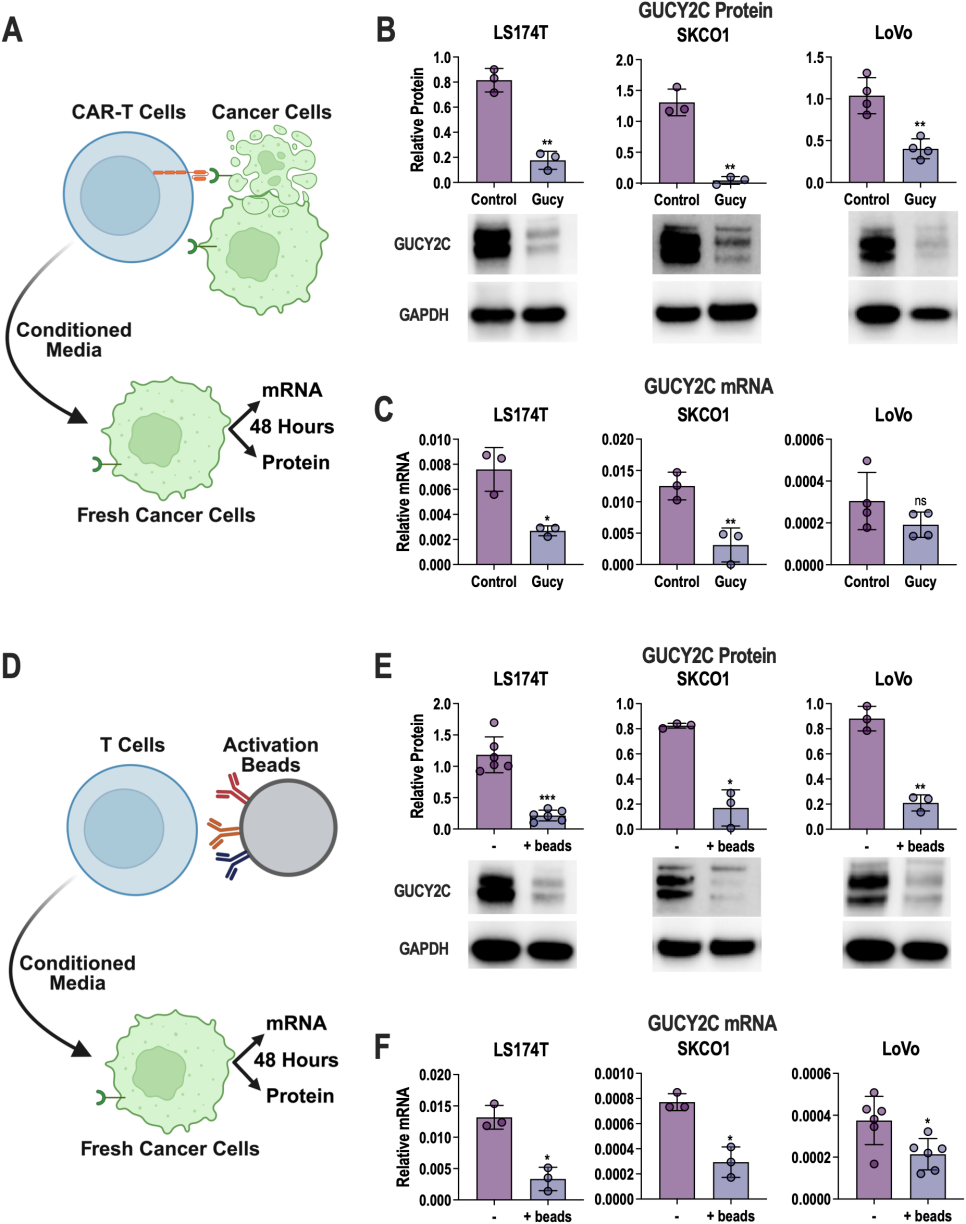
CART-conditioned media induce GUCY2C loss in low antigen CRC cells. **(A–F**) GucyCART cells were activated by 48 hour co-culture with T84 CRC cells **(A–C)** or untransduced T cells were activated with anti-CD3/CD2/CD28 beads **(D–F)**. Conditioned media (CM) were then collected, and fresh LS174T, SKCO1, or LoVo cells were exposed to CM for 48 hours before quantification of GUCY2C protein **(B, E)** and mRNA **(C, F)**. Controls include control CART cells **(A–C)** or beads lacking anti-CD3/CD2/CD28 antibodies **(D-F)**. Each data point represents the average of biological replicates in separate experiments (N = 3–5 experiments). A paired T-test was used to compare the conditions; ns = *p* > 0.05, *p < 0.05, **p < 0.01, ***p < 0.001. Figure schematics were generated using BioRender.com.

Previous literature on CART-induced antigen loss mechanisms suggests that antigen loss depends on CART cells directly contacting tumor cells to impose selection and/or survival pressure (35, 36, 38, 40). Initially, we hypothesized that GucyCART-induced GUCY2C loss depends on CART contact with CRC cells. Thus, treating CRC cells with media conditioned by activated GucyCART cells should not impact GUCY2C expression (**Figure 3A**). Unexpectedly, GUCY2C protein levels were drastically reduced by conditioned media from CRC + GucyCART cocultures (**Figure 3B**). GUCY2C mRNA levels were also significantly reduced in LS174T and SKCO1 cells, but not LoVo, which has very low GUCY2C mRNA expression at baseline (**Figure 3C**). To determine if this reduction reflects a generalizable product of activated human T cells, rather than a product of dying cancer cells or antigen activation of GucyCART, we tested conditioned media from anti-CD2/CD3/CD28 antibody-conjugated bead-activated human T cells lacking a CAR (**Figure 3D**). Indeed, GUCY2C protein (**Figure 3E**) and mRNA (**Figure 3F**) levels in all three CRC cell lines were substantially reduced by media from activated human T cells.

### 2.3 GUCY2C loss is mediated by the IFNγR-JAK-STAT1 signaling axis

**Figure 4:**
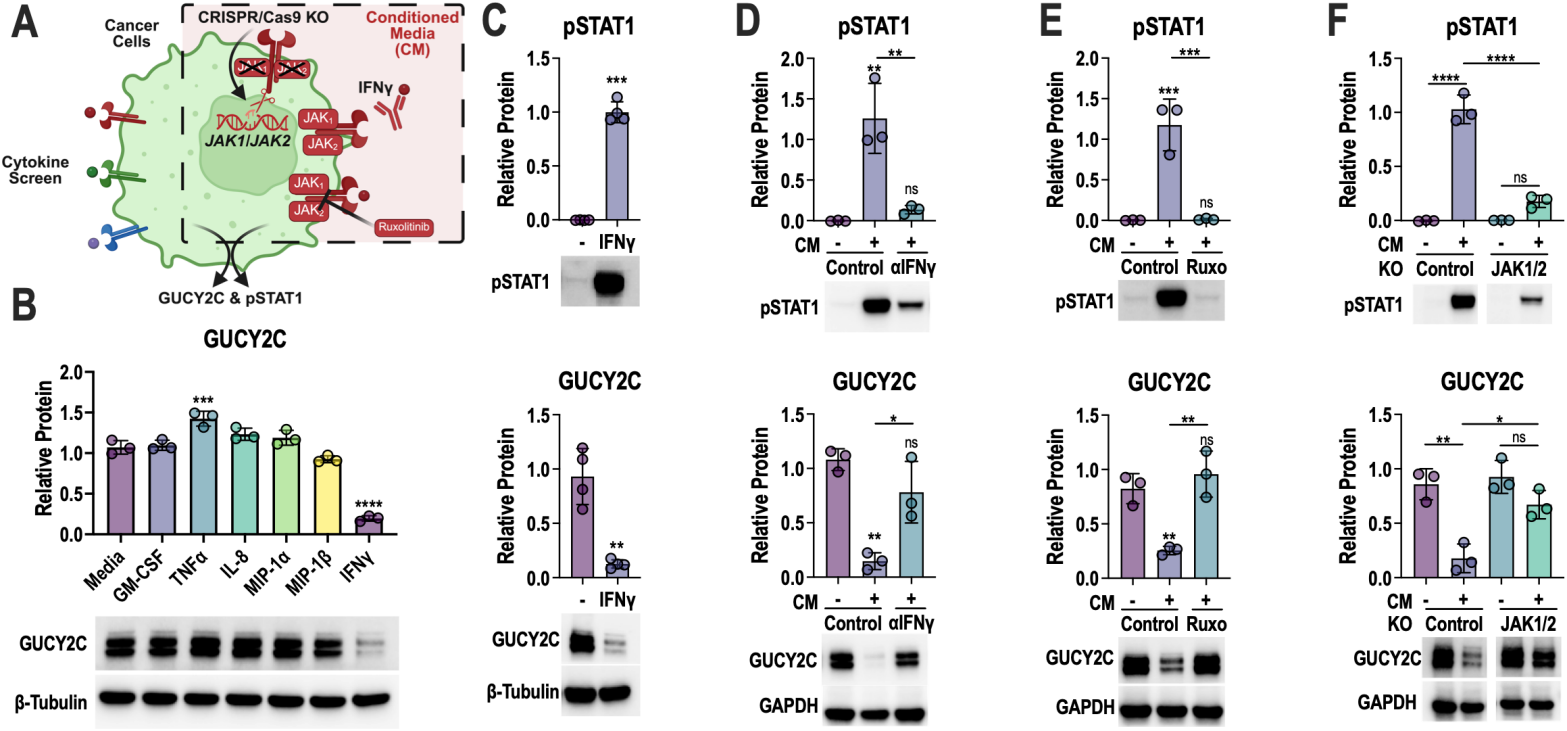
CART-induced GUCY2C loss is mediated by the IFNγ-JAK-STAT1 signaling axis. **(A)** LS174T cells and orthogonal approaches of cytokine screening **(B)** and confirmation **(C)**, IFNγ neutralizing antibody **(D)**, pharmacologic JAK1/2 blockade with ruxolitinib **(E)**, and JAK1/2 genetic knockout **(F)** were used to define the role of the IFNγ-JAK-STAT signaling axis in GUCY2C loss. **(B)** LS174T cells were treated with GM-CSF (20 ng/mL), TNFα (1 ng/mL), IL-8 (2 ng/mL), MIP-1α (2 ng/mL), MIP-1β (2 ng/mL), or IFNγ (15 ng/mL) for 48 hours, and GUCY2C protein levels were quantified. **(C)** LS174T cells were treated with 150 ng/mL IFNγ for 48 hours, and pSTAT1 and GUCY2C protein levels were quantified. **(D–F)** LS174T cells were treated with conditioned media (CM) for 48 hours from control or anti-CD3/CD2/CD28 bead-activated T cells, and pSTAT1 and GUCY2C protein levels were quantified; **(D)** 13 μg/mL anti-IFNγ neutralizing antibody was included in some conditions; **(E)** 2.5 μM ruxolitinib was included in some conditions; **(F)** LS174T cell pools previously treated with control CRISPR/Cas9 or JAK1 + 2 CRISPR/Cas9 were used. Each data point in **(B–F)** represents average of biological replicates from separate experiments (N = 3–4 experiments). In **(C–F)**, pSTAT, GUCY2C, and housekeeping control were examined on the same blot. The housekeeping protein is shown only below GUCY2C. A paired t-test was used to compare the two conditions in **(C)**; in **(B)** and **(D–F)**, one-way ANOVA was used to compare each condition to the control treatment, and additional comparisons are indicated by bars; ns = *p* > 0.05, *p < 0.05, **p < 0.01, ***p < 0.001, ****p < 0.0001. Figure schematics were generated using BioRender.com.

To further investigate the mechanism of GUCY2C loss, we employed cytokine screening, blocking antibody, pharmacologic, and gene knockout strategies (**Figure 4A**). The candidate list of cytokines secreted by activated T cells whose receptors are expressed by the CRC cancer cell lines included GM-CSF, TNFα, IL-8, MIP-1α, MIP-1β, and IFNγ. Only IFNγ recapitulated the effect of GucyCART and reduced total GUCY2C protein (**Figure 4B**) and surface expression of GUCY2C (**Supplementary Figure 1**). IFNγ receptor (IFNγR) is a heterodimer of IFNGR1 and IFNGR2 (44). Upon IFNγ binding, IFNGR1 and IFNGR2 dimerize, leading to transphosphorylation of JAK1 and JAK2, two Janus kinases associated with IFNGRs (45, 46). Phosphorylated JAK1 and JAK2 can then phosphorylate STAT1 (pSTAT1), which dimerizes and relocates to the nucleus to regulate gene expression (47). One of the genes upregulated by phosphorylated STAT1 is STAT1 itself creating a positive-feedback loop. As expected, treating LS174T cells with either IFNγ or T-cell-conditioned media induced STAT1 phosphorylation (**Figures 4C, D**), total STAT1 upregulation (**Supplementary Figure 2**) and GUCY2C loss (**Figures 4C, D**). Furthermore, IFNγ-neutralizing antibodies blocked the phosphorylation of STAT1 and upregulation of total STAT1, as well as prevented conditioned media-induced loss of GUCY2C (**Figure 4D**; **Supplementary Figure 2B**). To confirm the role of JAK1/2 in GUCY2C loss, we employed the well-established (48, 49) JAK1/2 inhibitor ruxolitinib (**Figure 4E**) and CRISPR-Cas9-mediated JAK1/2 elimination (**Figure 4F****;****Supplementary Figure 3**). Indeed, supplementing T-cell-conditioned media with ruxolitinib prevented STAT1 phosphorylation and loss of GUCY2C (**Figure 4E**). CRISPR-Cas9 knockout reduced JAK1/2 proteins ≥70% (**Supplementary Figure 3**), reduced STAT1 phosphorylation >80% (**Figure 4F**), and restored GUCY2C in the presence of T-cell-conditioned media (**Figure 4F**). Together, these data demonstrated that IFNγ is necessary and sufficient to reduce GUCY2C levels in the presence of activated GucyCART cells, mediated by the IFNγR-JAK1/2 signaling axis.

### 2.4 IFNγ-induced GUCY2C loss is mediated by cellular stress signaling

**Figure 5:**
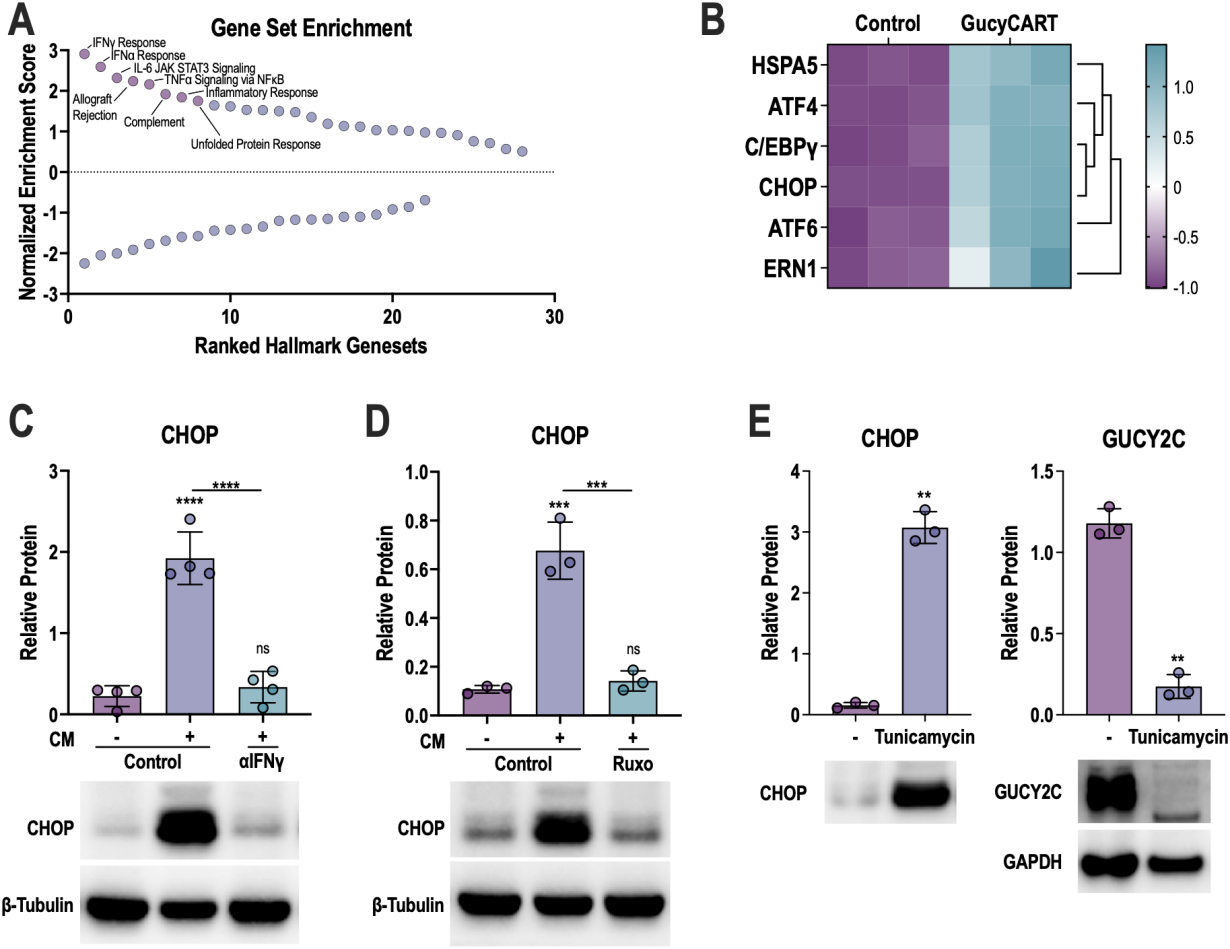
IFNγ-induced GUCY2C loss is mediated by cellular stress signaling. **(A)** Gene Set Enrichment Analysis (GSEA) of significantly up- and down-regulated Hallmark pathways in RNAseq data from LS174T cells treated with conditioned media (CM) from T84 co-cultures with control or GucyCART. **(B)** Significantly upregulated stress pathway-related genes from **(A)**. **(C, D)** LS174T cells were treated for 48 hours with CM ± 13 μg/mL anti-IFNγ neutralizing antibody (αIFNγ, **C**) or 2.5 μM ruxolitinib (Ruxo, **D**) and CHOP protein was quantified. **(E)** LS174T cells were treated with control or 2 μg/mL tunicamycin for 48 hours, and CHOP and GUCY2C proteins were quantified. Each data point in **(C–E)** represents the average from biological replicates in separate experiments (N = 3-4). In **(E)**, CHOP and GUCY2C were examined on the same blot and normalized to the GAPDH control (shown only below GUCY2C). One-way ANOVA was used to compare each condition in **(C)** and **(D)**, and a paired T-test was used to compare conditions in **(E)**; ns = *p* > 0.05, **p < 0.01, ***p < 0.001, ****p < 0.0001. Figure schematics were generated using BioRender.com.

To understand how IFNγR-JAK signaling induces GUCY2C loss, we conducted transcriptomic analyses of LS174T cells in the presence of control or CART-conditioned media, confirming the expected upregulation of the IFNγ response and other effector cytokine-related pathways, such as IFNα, IL-6-JAK-STAT, and TNFα (**Figure 5A**). Moreover, we found that the unfolded protein response (UPR) pathway was also significantly upregulated (**Figure 5A**). UPR is a crucial cellular signaling mechanism that can regulate protein production when cells are under inflammatory stress (50–53). Canonically, when misfolded and unfolded proteins accumulate in the endoplasmic reticulum (ER) to cause ER stress, these misfolded and unfolded proteins compete with HSPA5 to bind three well-defined sensor proteins: inositol-requiring enzyme 1 (ERN1), activating transcription factor 6 (ATF6), and protein kinase RNA-like ER kinase (PERK) (52, 54). These sensor proteins activate downstream signaling pathways involving ATF4, CCAAT/enhancer-binding protein gamma (C/EBPγ), and C/EBP homologous protein (CHOP), to restore normal ER functions by enhancing protein folding capacity, degrading misfolded proteins, and attenuating protein production (50, 55–57). Multiple key regulators of this stress signaling, including HSPA5, ATF4, ATF6, ERN1, CHOP, and C/EBPγ, were upregulated by T-cell-conditioned media (**Figure 5B**). We confirmed at the protein level that T-cell-conditioned media induces CHOP upregulation (**Figures 5C, D**), which was abolished by IFNγ-neutralizing antibodies (**Figure 5C**) and JAK/1/2 inhibition with ruxolitinib (**Figure 5D**). Moreover, treating LS174T cells with tunicamycin, a known chemical inducer of UPR (58–61), increased CHOP and reduced GUCY2C protein levels (**Figure 5E**).

### 2.5 4-phenylbutyrate rescues IFNγ-induced GUCY2C loss and allows for more complete CRC elimination

**Figure 6:**
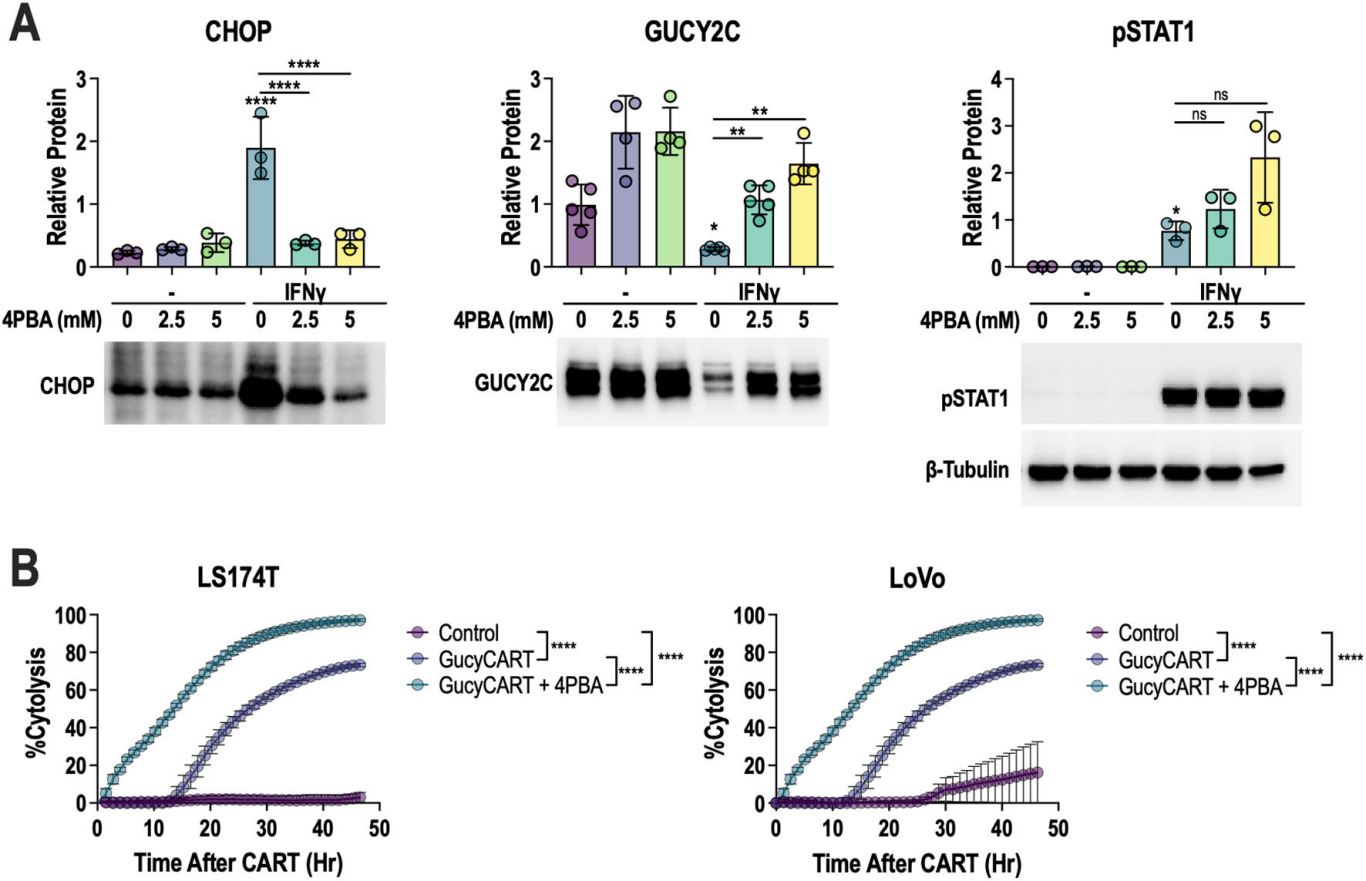
4PBA rescues IFNγ-induced GUCY2C loss and permits complete cytolysis. **(A)** LS174T cells were treated with control or 15 ng/mL IFNγ ± 2.5 or 5 mM 4PBA for 48 hours before quantification of CHOP, GUCY2C, and pSTAT1 proteins. Each data point represents the average of biological replicates in separate experiments (N = 3–5 experiments). In each experiment, analytes were examined on the same blot and normalized to the β-tubulin control (shown only below pSTAT1). One-way ANOVA was used to compare conditions to the control, and bars indicate other comparisons; ns = p > 0.05, *p < 0.05, **p < 0.01, ****p < 0.0001. **(B)** LS174T or LoVo cells were treated with control or GucyCART at E:T of 1:1 ± 2.5 mM 4PBA. Each data point represents the mean ± SD with n ≥ 3 technical replicates; representative of two experiments. AUCs were calculated for each condition, and one-way ANOVA was used for comparisons; ****p < 0.0001. Figure schematics were generated using BioRender.com.

The above data suggest that GucyCART-produced IFNγ induces GUCY2C loss by UPR induction, and UPR reversal might restore GUCY2C and improve *in vitro* CART function. In that context, 4-phenylbutyrate (4PBA) is a small-molecule chemical chaperone that alleviates protein misfolding stress in the ER and can reduce downstream activation of stress response pathways (62–65). Indeed, 4PBA reduced CHOP and restored GUCY2C protein, without altering canonical IFNγR/JAK/STAT1 signaling (**Figure 6A**). Furthermore, while 4PBA alone does not induce cell death (**Supplementary Figure 4**), 4PBA co-treatment improved the speed (time to 50% killing) and completeness (peak cytolysis) of LS174T and LoVo cell cytolysis (**Figure 6B**).

## 3 Discussion

A density of surface antigen sufficient to induce CART cell activation is a crucial parameter for CART efficacy (39, 66). CART requires substantially higher antigen levels to induce downstream effector functions compared to the threshold required for native T-cell receptor and peptide-MHC interaction (67, 68). Moreover, antigen heterogeneity in solid, compared to liquid, tumors further challenges CART activation (69, 70). In addition, previous studies have observed antigen reduction in clinical trials for both liquid and solid malignancies (20, 35, 37, 40–43). Here, we observed suboptimal efficacy of GucyCART in models of CRC with low GUCY2C levels and extended our *in vivo* observation *in vitro* to study the underlying mechanism. Here, we found that CRC cell lines with low/moderate GUCY2C levels lose antigen in the presence of GucyCART and identified an unexpected mechanism of antigen loss in “bystander” CRC cells driven by CART-produced IFNγ. Secreted IFNγ induced activation of JAK1/2, subsequent phosphorylation of STAT1, and activation of the UPR, leading to GUCY2C loss in bystander CRC cells, without direct contact between the tumor cell and GucyCART.

IFNγ has well-established benefits in T-cell immunity (71). Landmark studies in the early 2000s revealed the critical role of the IFNγ signaling axis in mouse models of “cancer immunosurveillance,” revitalizing the field of cancer immunology (72, 73). As a pivotal effector cytokine, IFNγ boosts T-cell immune functions by binding IFNγR on both T cells and tumor cells. IFNγ promotes cytotoxic T-cell differentiation, stimulates the release of perforin and granzymes, as well as secretion of chemokines to recruit additional T cells and other immune cells (71). In cancer cells, IFNγ upregulates surface peptide-MHC complexes, antigen-processing machinery, and adhesion molecules to enhance T-cell recognition (74). In the context of CART, IFNγ secretion has been linked to cytokine release syndrome (CRS) in patients (75, 76). However, IFNγR signaling in solid tumor cells is required for CART efficacy due to its roles in upregulating adhesion molecules and enhancing synapse formation (77). Previous research on tumor initiation and progression, cancer vaccines, and tumor-infiltrating T cells (TILs) has demonstrated contributions of IFNγ to reduced antigen levels, such as melanosomal antigens in melanoma (78), the endogenous retroviral antigen gp70 in murine CRC (79), HER2 in breast cancer (80), and annexin 2 in prostate cancer (81). Mechanisms revealed by those studies include transcription downregulation (78, 79) and modulation of the ubiquitin-proteasomal pathway (80, 81). Here, we identified a negative effect of IFNγ in reducing antigen levels in bystander CRC cells, in the context of GucyCART. However, while ruxolitinib blocked IFNγ-induced GUCY2C loss (**Figure 4**), it had a negative overall impact on GucyCART efficacy (**Supplementary Figure 5**), reflecting the previously identified impact on synapse formation (77), necessitating the identification of novel, targetable mechanisms underlying IFNγ-induced antigen loss.

The inability of ruxolitinib to improve efficacy while blocking antigen loss prompted us to further dissect the linkage between IFNγ/JAK and antigen reduction. Transcriptomic pathway analysis (**Figures 5A, B**), CHOP protein validation (**Figures 5C, D**), as well as the alleviation of CHOP (**Figures 5C, D**) and the rescue of GUCY2C with anti-IFNγ neutralizing antibody (**Figure 4D**), ruxolitinib (**Figure 4E**), and 4PBA (**Figure 6A**) strongly support the activation of stress signaling by IFNγ and the role of this pathway in antigen reduction. However, it is essential to distinguish between stress signaling and stress. High protein production and processing load induced by STAT1-driven transcription can lead to ER stress, subsequently activating stress signaling pathways (82, 83). Alternatively, phosphorylated JAK/STAT1 can directly activate stress sensors to initiate stress signals, even in the absence of stress (84, 85). Enrichr (86), a tool that integrates genome-wide ChIP experiments from ENCODE (87) and ChEA (88) projects, was used to investigate whether the regulation of specific transcription factors was altered in LS174 cells exposed to CART-conditioned media. The results indicate the IRF family members IRF8 and IRF1 were the top two enriched transcriptional regulators for the up-regulated genes (**Supplementary Figure 6**). Subsequently, using HOMER (89), we identified regulatory motifs that were enriched in the promoters of the up- and down-regulated genes. Motifs linked to IRF family members were among the top-enriched motifs in the up-regulated gene set (**Supplementary Figure 6**). Motif enrichment analysis of transcriptomic data from conditioned-media-treated LS174T cells revealed an enrichment for IRF1 and IRF8 motifs, supporting the activation of canonical IFNγ downstream signaling (90). In contrast, downregulation of E2F4 transcriptional targets and motifs for TATA-Box (91), KLF3 (92), TEAD1 (93), and HOXA9 (94) (**Supplementary Figure 6**) indicate global suppression of canonical transcription initiation and a stress-adapted state. Therefore, we speculate that UPR-induced antigen loss compromises transcription and translation in the stress-adapted state. Future studies are needed to dissect the mechanisms underlying CART-induced stress signals, mediated by IFNγ signaling downstream of the UPR, in bystander CRC cells.

GUCY2C is an intriguing therapeutic target being examined in multiple ongoing clinical trials of different therapeutic modalities. A recent phase I clinical trial evaluated the safety and preliminary antitumor activity of GCC19CART in 15 patients with metastatic CRC (33). GCC19CART employed the “CoupledCAR” platform that pairs GUCY2C-directed CART with CD19-directed CART, as well as additional transgenes to express IFNγ, IL-6, and IL-12 (33). In this clinical trial, all side effects were self-limiting and manageable, and 4 of 7 patients treated with the high dose of GCC19CART experienced a partial response (33). Although the promising phase I trial results yielded a major pharmaceutical acquisition for continued clinical development, our observations of IFNγ-induced GUCY2C loss may provide meaningful insights to improve the efficacy of GUCY2C-directed CART. Addressing the IFNγ-induced loss of GUCY2C without compromising the IFNγR/JAK/STAT signaling beneficial to cytotoxicity has the potential to deepen tumor clearance (**Figure 6B**), prolong the durability of clinical responses, and increase the percentage of responding patients. GUCY2C also has been used as a therapeutic target for cancer vaccines. In a phase I trial involving 10 early-stage CRC patients, an adenovirus-based GUCY2C cancer vaccine produced promising safety and immunogenicity results (26). A phase II trial for the GUCY2C cancer vaccine is currently ongoing (NCT04111172). In contrast to our observations in the context of CART, we hypothesize that IFNγ-induced GUCY2C loss should not be detrimental for cancer vaccine efficacy. Although total GUCY2C protein is reduced in CRCs, GUCY2C peptide presentation by surface HLA should be boosted upon exposure to IFNγ, thus improving T-cell recognition.

Studies here focused mainly on GUCY2C and GucyCART. However, these observations might be generalized to other CART targets and other modalities of immunotherapy. Indeed, similar to GUCY2C, we observed total CDH17 and HER2 reduction upon IFNγ exposure (**Supplementary Figure 7**). Further, surface CDH17 and HER2 (measured by flow cytometry) were reduced by IFNγ (**Supplementary Figure 7**), aligning with the surface antigen requirement of CAR-T cells. In contrast, some other targets, including IL13Rα2, EGFR, and mesothelin, were reported to remain unchanged following IFNγ exposure (77). It is unclear why some targets are sensitive to IFNγ while some are resistant. Further studies on the structures of these transmembrane proteins or their synthesis processes may provide valuable insights for strategies to overcome the IFNγ-induced antigen loss. In addition, IFNγ-induced antigen loss is likely to pose challenges to other therapies targeting native surface antigen, including CD3 bispecific antibodies (BiTEs). In contrast, T-cell receptor (TCR)-engineered T-cell therapies, which target peptide-HLA complexes, may be insensitive, similar to our expectations for GUCY2C-directed cancer vaccines. Thus, immunotherapies targeting native antigen, such as CART and BiTEs, but not peptide-HLA complexes, such as TCR-T cells and vaccines, might be impacted by IFNγ-induced antigen reduction.

To our knowledge, this is the first study to demonstrate that IFNγ causes CART antigen loss on cancer cells. This novel mechanism of antigen loss, unlike many well-established antigen escape mechanisms (35, 36, 38, 39, 66, 95, 96), does not require genetic alterations or for cancer cells to come into direct contact with CART cells to reduce antigen. In the context of CART and BiTE therapies, the reduction of antigen in bystander cells due to IFNγ secreted by stimulated T cells can be a barrier to the complete elimination of tumors. Given the overall positive effects of IFNγ on T-cell-mediated cytotoxicity, we have further identified cellular stress signaling as the key mediator linking the IFNγ/JAK/STAT pathway to the observed reduction in antigen. Although 4PBA alleviates cell stress signaling and preserves phosphorylation of STAT1 in the presence of IFNγ *in vitro*, *in vivo* application of 4PBA is challenging. A previous study demonstrated that 4PBA has a short half-life of <30 minutes in mice (97). This pharmacokinetic characteristic renders 4PBA a poor candidate for blocking GUCY2C loss and enhancing GucyCART efficacy in animal models or patients. Continued investigation of mechanisms underlying IFNγ-induced stress responses, defining the mechanisms by which stress signaling reduces the CRC target antigens GUCY2C, CDH17, and HER2, and exploring the impacts of antigen structure and synthesis on differential sensitivity to IFNγ-induced loss, are necessary to develop therapeutic strategies to block IFNγ-induced antigen loss, preserve IFNγ-induced synapse formation, and enhance the efficacy of CRC CART therapy for patients.

## 4 Methods

### 4.1 Lentivirus plasmid production

Cassettes for CAR transfer plasmids (30) were synthesized and cloned (GenScript Biotech, Piscataway, NJ) into the pCDH-EF1α-MCS-T2A-GFP lentiviral transfer vector (CD525A-1, System Biosciences). All plasmids, including lentiviral packaging and envelope plasmids, were transformed into NEB Stable Competent *E. coli* (C3040H, New England Biolabs). *E. coli* were cultured in LB Broth, Miller (BP1426-2, Fisher Scientific) supplemented with 1.9% Bacto Yeast Extract (212750, Thermo Fisher Scientific) and 100 μg/mL Ampicillin (A8351-5G, Sigma-Aldrich). DNA was purified from overnight bacterial cultures using the Purelink Expi Endotoxin-Free Maxi Plasmid Purification Kit (A31231, Thermo Fisher Scientific). DNA pellets were resuspended in Endotoxin-free water to a final concentration of ~ 1 μg/μL.

### 4.2 Lentivirus production

T-225 flasks were coated with 5 μg/cm^2^ poly-d-lysine (354210, Corning), washed with DPBS (21-031-CV, Corning), and allowed to dry before 28.4 million HEK293T/17 cells (CRL11268, American Type Culture Collection (ATCC)) were seeded in cell culture medium consisting of Advanced DMEM (12491023, Thermo Fisher Scientific) supplemented with 5% heat-inactivated FBS (A38400-01, Gibco) and 1X GlutaMAX (35050-061, Gibco). The next day, Lipofectamine 3000 transfection reagent (L3000150, Thermo Fisher Scientific) was used to deliver the lentiviral plasmids in the following amounts: 17.7 μg CAR transfer plasmid, 14.7 μg pRSV-Rev (12253, Addgene), 31 μg pMDLg/pRRE (12251, Addgene), and 7.6 μg pMD2.G (12259, Addgene) in Advanced DMEM. A complete media exchange was performed 6 hours post-transfection. Media was collected at 24 and ~52 hours post-transfection and filtered using a 0.45 μm αPES filter unit (09-740-63E, Fisher Scientific). Lentivirus was concentrated using a 4X Polyethylene Glycol 8000 (BP233-1, Fisher Scientific) solution that was incubated on a wave rotator at 4 °C overnight, followed by centrifugation at 1600×g for 1 hour at 4 °C. Lentiviral pellets were resuspended at a 200X concentration in lentivirus storage buffer: 10 mM Tris, pH 7.4 (648315–100 ML, EMD Millipore), 10% lactose (61339–25 G, Sigma-Aldrich), 25 mM Proline (81709–10 G, Sigma-Aldrich) in DPBS. The vector was stored at -80 °C. Lentivirus titer was determined by the percent of GFP-positive HEK293T/17 cells transduced in the presence of 0.8 μg/mL Polybrene (TR- 1003-G, Millipore Sigma), using a BD FACSymphony A5 or A3 SORP Flow Cytometer.

### 4.3 CAR-T cell production

T cell culture medium was composed of RPMI-1640 (10-041-CV, Corning) supplemented with 10% heat-inactivated FBS (A38400-01, Gibco), 1X insulin-transferrin-selenium (ITS-G 41,400-045, Gibco), 10 mM N-acetyl-L-cysteine (A9165, Millipore Sigma), 1X GlutaMAX (35050-061, Gibco), 1X glucose solution (A24940-01, Gibco), 1X sodium pyruvate (11360-070, Gibco), 1X MEM non-essential amino acids (11140-050, Gibco), 1X HEPES buffer (15630-080, Gibco), 1X penicillin- streptomycin (15140-122, Gibco), 55 μM 2-mercaptoethanol (21985-023, Gibco), and two cytokines: 10 ng/mL human IL-7 and 10 ng/mL human IL-15 (BRB Preclinical Biologics Repository, NCI Biological Resources Branch, Frederick, MD). Primary human T cells were magnetically sorted from the peripheral blood of three healthy donor leukopaks (130-096-535, Miltenyi Biotec or 200-0092, Stemcell Technologies). T cells (10^6^ cells/mL) were activated at a 1:1 ratio with anti-CD3/CD28/CD2 magnetic beads (130-091-441, Miltenyi Biotec) in T cell culture medium. One day after activation, T cells were transduced with CD19-directed CAR (control) or GucyCAR lentivirus at an MOI of 5 with 0.8 μg/mL polybrene (TR-1003-G, Millipore Sigma). At least one well of T cells was left untransduced. Three days after activation, activation beads were removed, and T cells were transferred to G-Rex plates (80660 M, Wilson Wolf). Cytokines were replenished every two to three days. On day 12 after activation, T cell concentration and viability were assessed using the Guava MUSE Cell Analyzer (Cytek Biosciences). The percentage of CAR-positive T cells (using the GFP reporter) was measured using a BD FACSymphony A5 or A3 SORP Flow Cytometer. Untransduced T cells were added to control and GUCY2C-directed CAR-T cells to produce equal percentages of GFP-positive (transduced) T cells. CAR-T cells were cryopreserved using CryoStor CS10 (07930, Stemcell Technologies) at a density of 20–30 million cells/mL.

### 4.4 CRC cell lines

LS174T (CL-188, ATCC), SKCO1 (HTB-39), LoVo (CCL229, ATCC), and T84 (CCL-248, ATCC) were obtained ATCC. Cell identities were confirmed by STR profiling. LS174T cells were cultured in Eagle’s Minimal Essential Medium (10- 010-CV, Corning) supplemented with 10% fetal bovine serum (35-010-CV, Corning). SKCO1 cells were cultured in Eagle’s Minimal Essential Medium (10-010-CV, Corning) supplemented with 10% fetal bovine serum (35-010-CV, Corning), 1X GlutaMAX (35050-061, Gibco), 1X NEAA (11140-050, Gibco), 1X sodium pyruvate (11360-070, Gibco), and pH adjusted to 7.5. LoVo cells were cultured in F-12K medium (10-092-CV, Corning) supplemented with 10% FBS (35-010-CV, Corning). T84 cells were cultured in Advanced DMEM/F-12 50%/50% (12634028, Thermo Fisher Scientific) supplemented with 5% FBS (35-010-CV, Corning).

### 4.5 Conditioned media production

For CART-conditioned media, T84 cells (0.3x10^6^) were plated in 1.5 mL of T84 culture medium per well in a 6-well plate. The next day, 0.1x10^6^ control or GucyCART cells were added in 500 μL RPMI-1640 per well. For T-cell-conditioned media (no CARs), primary human T cells were activated and cultured as above for CART manufacturing, but were left untransduced. On day 12, 10^6^ cells were activated at a 1:1 ratio with anti-CD3/CD28/CD2 magnetic beads in RPMI-1640 supplemented with 10% heat-inactivated FBS. After 48 hours of co-culture (CART with T84 or untransduced T cells with beads), conditioned medium was collected, centrifuged at 1600 rpm for 5 minutes, and then filtered through a 0.45 μm PVDF syringe filter (Millipore, SLHVR33RS). Conditioned media were aliquoted and stored at ≤ -20 °C.

### 4.6 mRNA isolation and analysis

Cells were lysed with Trizol, and RNA was purified with the Direct-zol RNA miniprep (Zymo Research, R2053). RNA concentration and purity were analyzed with a Nanodrop 1000 (Thermo Fisher Scientific). Complementary DNA was synthesized using the TaqMan Reverse Transcription kit according to the manufacturer’s instructions (N8080234, Thermo Fisher Scientific). Transcripts were quantified by qRT-PCR using Taqman primer probes (Human GUCY2C Assay ID Hs00990106_m1, Thermo Fisher Scientific; Human GAPDH Assay ID Hs02758991_g1, Thermo Fisher Scientific) on a QuantStudioTM 3 Real-Time PCR System (Thermo Fisher Scientific), with TaqMan Universal PCR Master Mix (4318157, Thermo Fisher Scientific). RNA selection and sequencing were performed by Azenta (Burlington, MA). Poly(A) selection was performed using NEBNext Ultra II RNA Library Prep Kit for Illumina (San Diego, CA). Libraries were sequenced on an Illumina NovaSeq X platform, generating 150-bp paired-end reads. Trimming, alignment, and read counting were performed with in-house Python scripts implemented in Snakemake (98). Raw reads were processed with Trimmomatic (99) to remove low-quality reads, including adapter sequences. Reads were aligned to the human (GRCh38) reference genome using STAR (v2.7.11b) (100), and reads mapping to genes were quantified with RSEM (v1.3.3) (101) in unstranded mode. Differential gene expression was performed with DESeq2 (v1.42.1) (102) to compare LS174T cells treated with GucyCART-conditioned media versus cells treated with control CART-conditioned media. Differentially expressed genes (DEGs), ordered by log_2_(fold change), were used for preranked gene set enrichment analysis (GSEA) (103) with fgsea (v1.28.0) (104). The DEGs were compared to the Hallmark gene sets in the Human Molecular Signatures Database (MSigDB, v2024.1.Hs) (105). Software packages DESeq2 and fgsea were implemented in R (v4.3.1, 2023). Transcription factor and motif enrichment studies were conducted using Enrichr (86) and HOMER (89), respectively.

### 4.7 Protein isolation and immunoblot analysis

Cells were washed once with cold DPBS and lysed with Radio-Immunoprecipitation Assay (RIPA) buffer supplemented with HALT Protease and Phosphatase Inhibitor cocktail (78441, Thermo Fisher Scientific). After shaking for 15–20 minutes on ice, the contents were pipetted vigorously 10 times and transferred to centrifuge tubes. Tubes were centrifuged at 16,000 g for 15 minutes. Supernatants were transferred to new microcentrifuge tubes and diluted with 4X Invitrogen NuPage LDS Sample Buffer (NP0007, Fisher Scientific) supplemented with 10% 2-Mercaptoethanol (60-24-2, Millippore Sigma). Lysates were boiled for 2 minutes at 90 °C and stored at -20 °C. Samples were loaded into NuPage 4-12% Bis-Tris Protein Gels (NP0336BOX, Thermo Fisher Scientific) along with Invitrogen Novex Sharp Pre-Stained Protein Ladder (LC5800, Thermo Fisher Scientific). Gels were run at 135 V for 75 minutes. Gels were transferred to the iBlot 3 Transfer Stack, nitrocellulose (IB33002X3, Thermo Scientific). The membrane was blocked in 10% BSA in PBS with Tween-20 (PBS-T). Membranes were probed using an anti-human GUCY2C antibody (37517, Cell Signaling Technology), anti-human GAPDH (2118S, Cell Signaling Technology), anti-human STAT1 (14994T, Cell Signaling Technology), anti-human phospho-STAT1 (9167S, Cell Signaling Technology), anti-human CHOP (2895S, Cell Signaling Technology), anti-human β-actin (2128S, Cell Signaling Technology), anti-human EpCAM (2929S, Cell Signaling Technology) anti-human CDH17 (88594T, Cell Signaling Technology), and anti-human HER2 (2165T, Cell Signaling Technology). An HRP-conjugated Goat anti-Rabbit or anti-Mouse IgG secondary antibody (111-035046, 115-035-003, Jackson Immunoresearch) was used at 1:12,500 in 10% BSA in PBS-T. SuperSignal West Femto Chemiluminescent Substrate (34096, Thermo Fisher Scientific) was used to detect the bands on a ChemiDoc MP Image System (Bio-Rad Laboratories, Inc.). Images were analyzed using Image Lab, version 6.1.0 (Bio-Rad Laboratories, Inc.).

### 4.8 Flow cytometry

GUCY2C, CDH17, and HER2 surface levels were examined via flow cytometry. Cells were plated and treated accordingly. After treatment, cells were trypsinized and quenched with cold cell culture media. Cells were washed three times with cold PBS and incubated with human FC Block (BD Bioscience, 564219) on ice for 15 min to minimize non-specific antibody binding. Cells were then washed three times with FACS buffer and incubated with anti-GUCY2C clone 5F9 conjugated to PE (PE/R-Phycoerythrin Conjugation Kit; abcam, 102918), CDH17-PE (BPS Bioscience, 102827-2), or HER2-PE (BioLegend, 324406) antibodies on ice for 30 min. Cells were then washed three times in FACS buffer. Cells were then resuspended in FACS buffer supplemented with SYTOX Blue dead cells (Thermo Scientific S34857) and analyzed on a FACSymphony A3/A5 cytometer.

### 4.9 xCELLigence cytotoxicity assay

CART *in vitro* cytotoxicity was measured in real-time using the Agilent xCELLigence Real Time Cell Analysis (RTCA) analyzer (Agilent Technologies, Inc.). Culture media for each cell line were added to each well (100 μL/well) of an E-Plate 96 (5232368001, Agilent Technologies, Inc.) to establish a baseline impedance measurement for each well. Target cells were trypsinized (25-053- CI, Corning) and counted using a hemocytometer. Diluted in their culture media, 4x10^4^ LS174T or 2x10^4^ SKCO1 or LoVo cells were added to each well in 50 μL. The plate was rested at room temperature for 15 minutes before being placed into the xCELLigence RTCA machine. The cells were allowed to adhere overnight or until the cell index was above 1. Control or GucyCART cells were thawed and added in 50 μL RPMI-1640 (10-041-CV, Corning) at the desired effector to target ratio. The E-Plate was reinserted into the xCELLigence RTCA machine, and cell impedance measurements were collected every 15 minutes. The collected data were analyzed using RTCA Software Pro (Agilent Technologies, Inc.).

### 4.10 CytoTox-Glo Cytotoxicity Assay

Cells were plated in 96-well plates at 15,000 cells/well to adhere overnight in 50 μL of cell culture media. Cells were treated with 2.5 mM 4PBA or 2 μg/mL puromycin for the indicated times. Cell culture supernatant and cells analyzed using the CytoTox-Glo Cytotoxicity Assay (Promega, G9290). Viable cell luminescence was calculated by subtracting dead cell luminescence (supernatant) from the total cell luminescence.

### 4.11 CRISPR editing

SpCas9 protein (IDT, 1081058) and sgRNAs (JAK1: TCAGGTCATGCGTGGACACG and JAK2: TCCATATAGATGAGTCAACC) were purchased from Integrated DNA Technology. RNPs were produced with 120 pmol sgRNA and 104 pmol sgCas9, and incubated at room temperature for 10–20 minutes. LS174T cells (2x10^5^) were resuspended with 19 μL nucleofector solution SE with supplement (Lonza, V4XC-1032) and then combined with 5 μL RNP and 1 μL electroporation enhancer (IDT, 1075915). Cells were then electroporated using a Lonza 4D-Nucleofector X unit (AAF-1003X) with the DS-150 program. Electroporated cells were then allowed to recover and expand.

### 4.12 *In vivo* mouse models

NSG mice (JAX stock #005557) were injected intraperitoneally with 1x10^6^ luciferase-expressing LS174T or 2.5x10^6^ LoVo cells in 100 μL PBS. Control or GucyCART cells (5-6x10^6^ in 100 μL PBS) were administered via intravenous injection on day 14 after cancer cell inoculation. Tumor growth was monitored by subcutaneous injection of a 250 μL solution of 15 mg/mL D-luciferin potassium salt (Gold Biotechnologies) in PBS and imaging after 5–8 minutes of exposure using the Caliper IVIS Lumina-XR imaging station (Perkin Elmer). Total radiance (photons/second) was quantified using Living Image *In Vivo* Imaging Software (Perkin Elmer).

## Data Availability

The original contributions presented in the study are publicly available. The datasets supporting the conclusions of this article are available in LabArchives at https://dx.doi.org/10.25833/0fhw-bd62. RNA-seq data have been deposited in the NCBI Gene Expression Omnibus (GEO) under accession GSE322823 (https://www.ncbi.nlm.nih.gov/geo/query/acc.cgi?acc=GSE322823).
