# Supplementary material for: IFNγ-induced antigen loss in chimeric antigen receptor-T cell therapy
Source: Front Immunol. 2026 Mar 17;17:1772472. doi: 10.3389/fimmu.2026.1772472 (PMC13036235; doi:10.3389/fimmu.2026.1772472)
Supplement: Supplementary file 1 [file DataSheet1.pdf]

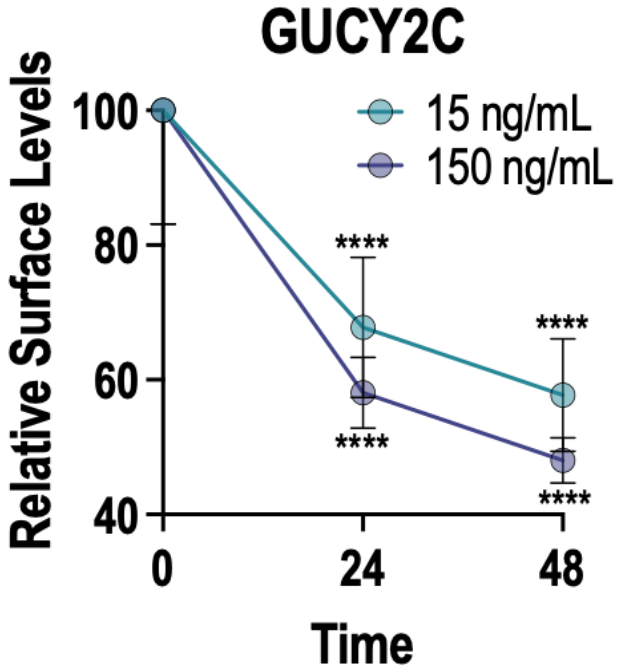

Supplementary Figure 1. IFNγ induces surface GUCY2C loss. LS174T cells were treated with 150 ng/mL or 15 ng/mL IFNγ for 24 or 48 hours. Flow cytometry was used to evaluate the surface levels of GUCY2C relative to starting levels (untreated control). N = 3 technical replicates; **** $p < 0.0001$, Two-way ANOVA.

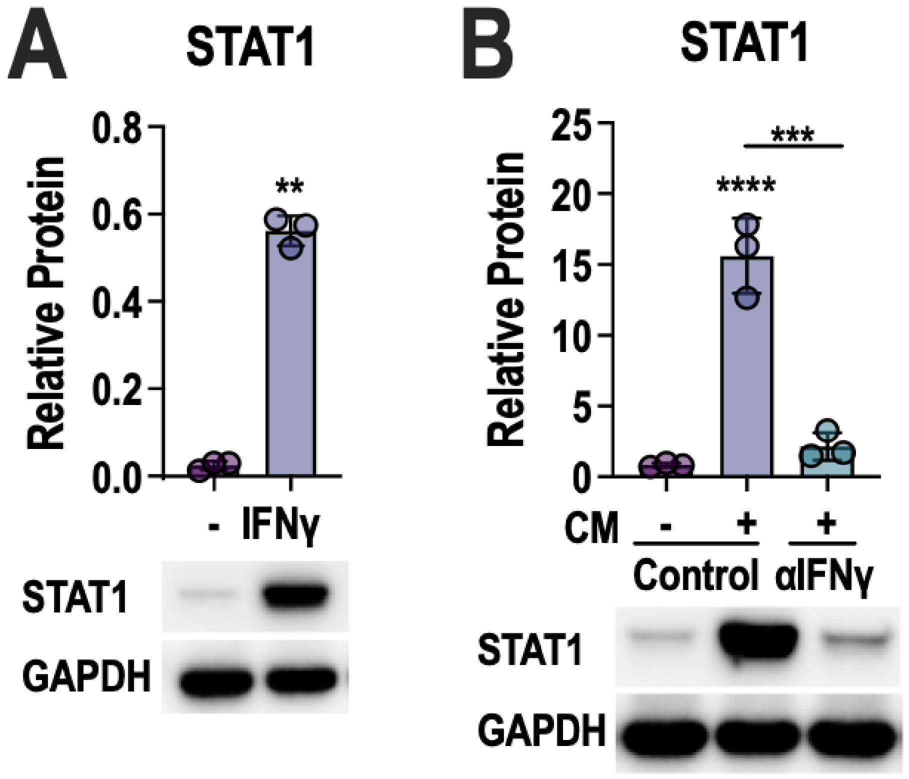

Supplementary Figure 2. IFNγ upregulates total STAT1 levels. A) LS174T cells were treated with 150 ng/mL IFNγ for 48 hours and total STAT1 protein levels were quantified. **B**) LS174T cells were treated with conditioned media (CM) for 48 hours from control or anti-CD3/CD2/CD28 bead-activated T cells and total STAT1 levels were quantified; αIFNγ indicates treatment of CM with 13 μg/mL anti-IFNγ neutralizing antibody. Each data point in **A** and **B** represents the average from a biological replicate (N = 3); ** $p < 0.01$, *** $p < 0.001$, **** $p < 0.0001$, paired T-test (**A**) or One-way ANOVA (**B**).

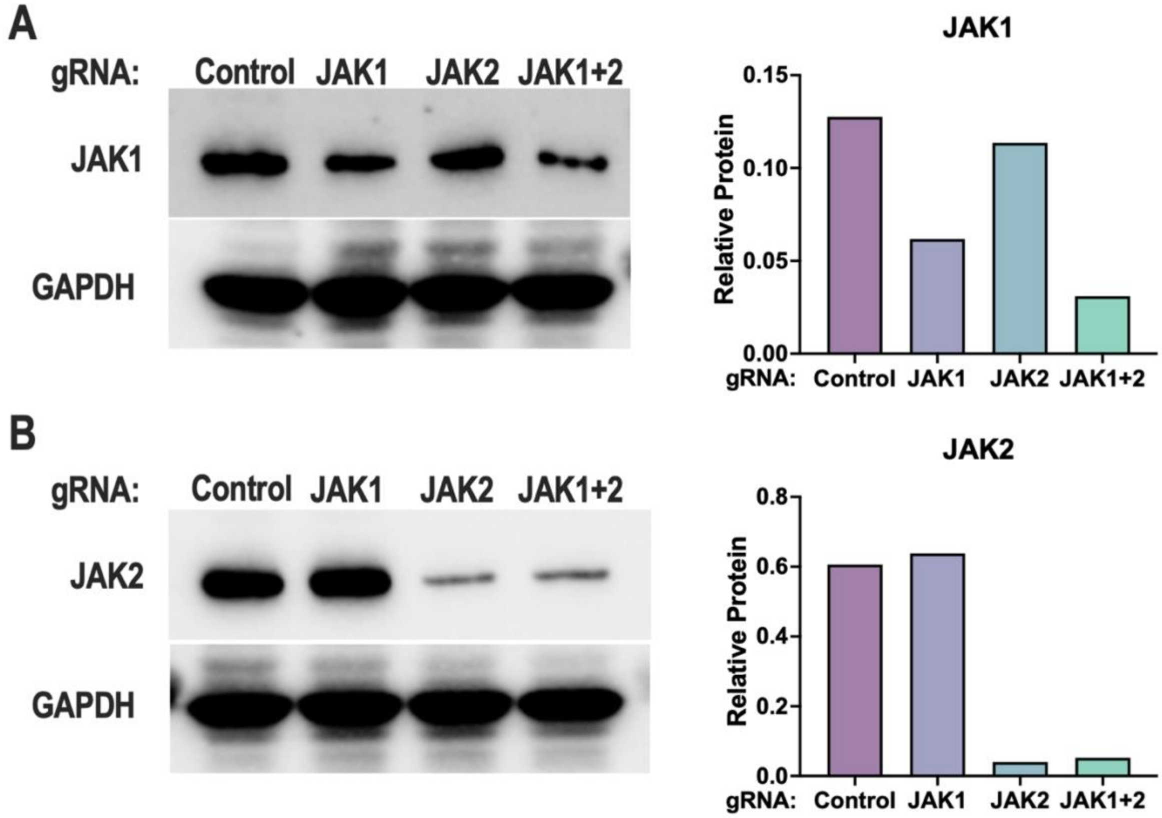

Supplementary Figure 3. JAK protein levels were reduced in JAK knockout pools. A-B) LS174T cells were electroporated with CRISPR/Cas9 RNPs produced with JAK1, JAK2, or JAK1+2 gRNAs or without gRNA (control). Relative protein levels of JAK1 (**A**) and JAK2 (**B**) were quantified.

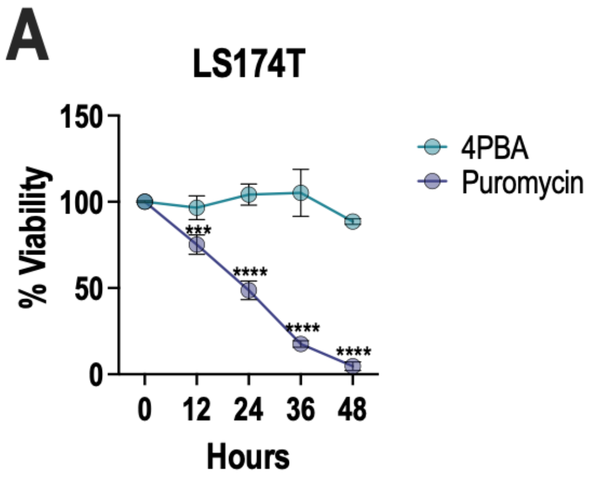

Supplementary Figure 4. 4PBA alone does not significantly negatively impact cell survival. LS174T cells (**A**) and LoVo cells (**B**) were treated with 2.5 mM 4PBA or 2 μg/mL puromycin (as a positive control) for up to 48 hrs. Cell viability was determined with a CytoTox-Glo Cytotoxicity Assay relative to untreated cells. N = 3 technical replicates; * $p < 0.05$, *** $p < 0.001$, **** $p < 0.0001$, Two-way ANOVA vs time 0.

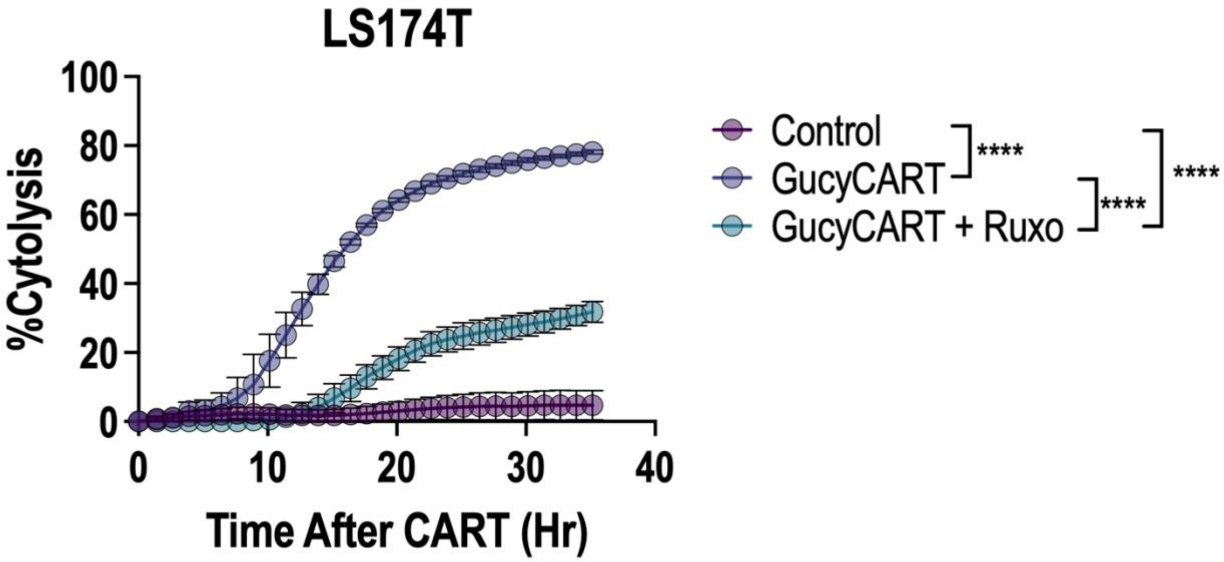

Supplementary Figure 5. Ruxolitinib reduces GucyCART efficacy. LS174T cells were treated with control or GucyCART ± 2.5 μM ruxolitinib. Area under the curve (AUC) was calculated for each condition, and one-way ANOVA was used for comparisons; **** p <0.0001.

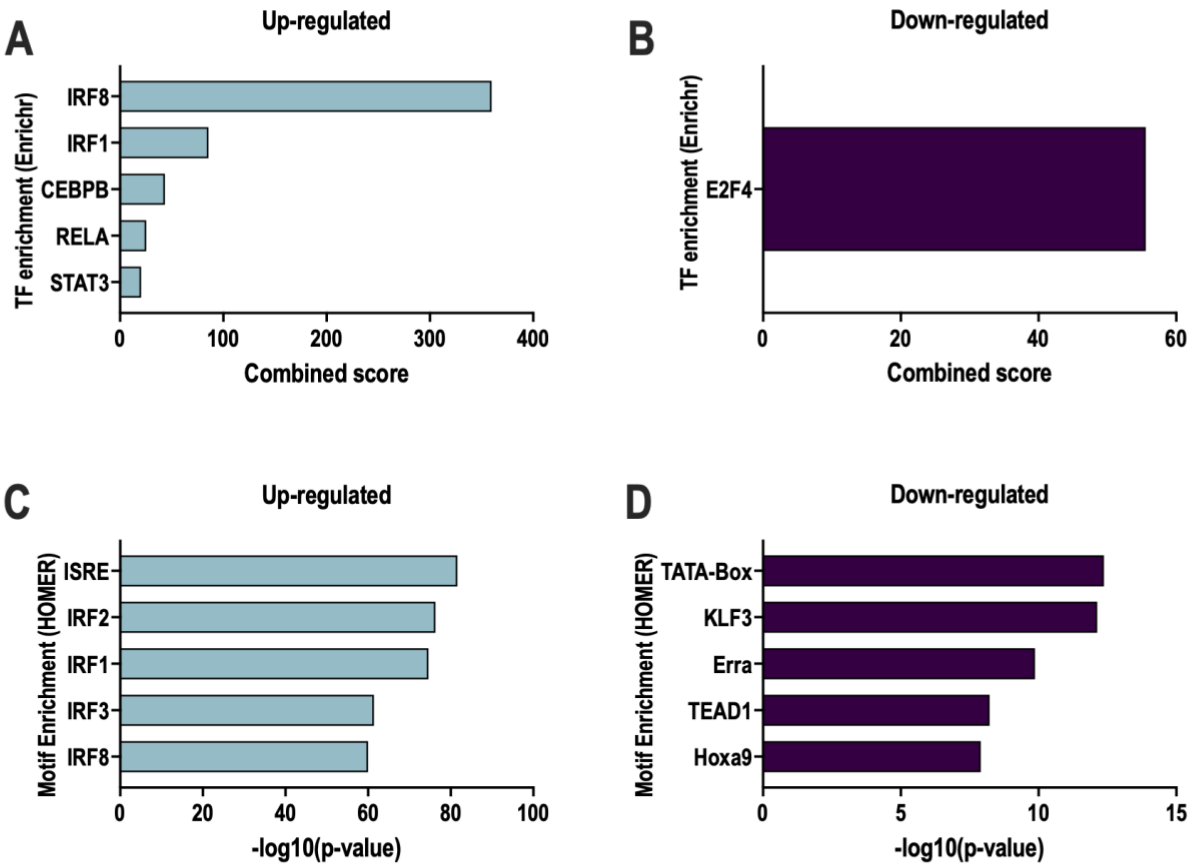

Supplementary Figure 6. Transcription factor analysis following CART-conditioned media exposure. Enrichr transcription factor (TF) enrichment of altered genes (**A,B**) and HOMER-calculated promoter motif enrichment of altered genes (**C,D**) analyses were conducted on RNAseq data from LS174T cells treated with conditioned media (CM) from LS174T co-cultures with control or GucyCART. The top five up- and down-regulated TF/motifs are shown, except for **B,** where only one transcription factor enrichment of genes was identified (adjusted $p < 0.05$).

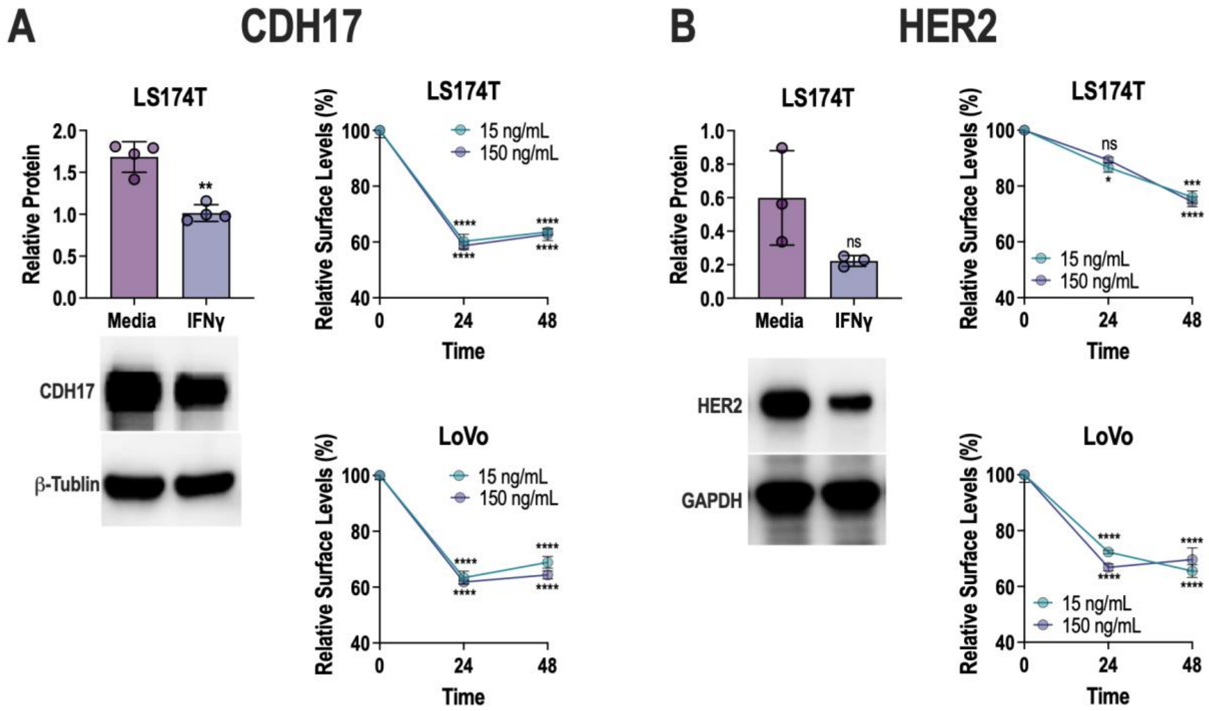

Supplementary Figure 7. Total and surface CDH17 and HER2 protein levels are reduced with IFNγ treatment. A-B, left) LS174T cells were treated with 150 ng/mL IFNγ for 48 hrs, and total CDH17 (**A**) and HER2 (**B**) protein were quantified by immunoblot (left). N = 3 technical replicates; ns = $p > 0.05$, ** $p < 0.01$, paired T-test. **A-B, right**) LS174T cells and LoVo cells were treated with 15 or 150 ng/mL IFNγ for 24 or 48 hrs. Flow cytometry was used to quantify surface levels of CDH17 and HER2 (right). N = 3 technical replicates; ns = $p > 0.05$, * $p < 0.05$, *** $p < 0.001$, **** $p < 0.0001$, Two-way ANOVA.
